# Age distribution, trends, and forecasts of under-5 mortality in 31 sub-Saharan African countries: A modeling study

**DOI:** 10.1371/journal.pmed.1002757

**Published:** 2019-03-12

**Authors:** Iván Mejía-Guevara, Wenyun Zuo, Eran Bendavid, Nan Li, Shripad Tuljapurkar

**Affiliations:** 1 Department of Biology, Stanford University, Stanford, California, United States of America; 2 Center for Population Health Sciences, Stanford University School of Medicine, Stanford, California, United States of America; 3 Primary Care and Population Health, Stanford University School of Medicine, Stanford, California, United States of America; 4 United Nations Population Division, New York, New York, United States of America; London School of Hygiene and Tropical Medicine, UNITED KINGDOM

## Abstract

**Background:**

Despite the sharp decline in global under-5 deaths since 1990, uneven progress has been achieved across and within countries. In sub-Saharan Africa (SSA), the Millennium Development Goals (MDGs) for child mortality were met only by a few countries. Valid concerns exist as to whether the region would meet new Sustainable Development Goals (SDGs) for under-5 mortality. We therefore examine further sources of variation by assessing age patterns, trends, and forecasts of mortality rates.

**Methods and findings:**

Data came from 106 nationally representative Demographic and Health Surveys (DHSs) with full birth histories from 31 SSA countries from 1990 to 2017 (a total of 524 country-years of data). We assessed the distribution of age at death through the following new demographic analyses. First, we used a direct method and full birth histories to estimate under-5 mortality rates (U5MRs) on a monthly basis. Second, we smoothed raw estimates of death rates by age and time by using a two-dimensional P-Spline approach. Third, a variant of the Lee–Carter (LC) model, designed for populations with limited data, was used to fit and forecast age profiles of mortality. We used mortality estimates from the United Nations Inter-agency Group for Child Mortality Estimation (UN IGME) to adjust, validate, and minimize the risk of bias in survival, truncation, and recall in mortality estimation. Our mortality model revealed substantive declines of death rates at every age in most countries but with notable differences in the age patterns over time. U5MRs declined from 3.3% (annual rate of reduction [ARR] 0.1%) in Lesotho to 76.4% (ARR 5.2%) in Malawi, and the pace of decline was faster on average (ARR 3.2%) than that observed for infant (IMRs) (ARR 2.7%) and neonatal (NMRs) (ARR 2.0%) mortality rates. We predict that 5 countries (Kenya, Rwanda, Senegal, Tanzania, and Uganda) are on track to achieve the under-5 sustainable development target by 2030 (25 deaths per 1,000 live births), but only Rwanda and Tanzania would meet both the neonatal (12 deaths per 1,000 live births) and under-5 targets simultaneously. Our predicted NMRs and U5MRs were in line with those estimated by the UN IGME by 2030 and 2050 (they overlapped in 27/31 countries for NMRs and 22 for U5MRs) and by the Institute for Health Metrics and Evaluation (IHME) by 2030 (26/31 and 23/31, respectively). This study has a number of limitations, including poor data quality issues that reflected bias in the report of births and deaths, preventing reliable estimates and predictions from a few countries.

**Conclusions:**

To our knowledge, this study is the first to combine full birth histories and mortality estimates from external reliable sources to model age patterns of under-5 mortality across time in SSA. We demonstrate that countries with a rapid pace of mortality reduction (ARR ≥ 3.2%) across ages would be more likely to achieve the SDG mortality targets. However, the lower pace of neonatal mortality reduction would prevent most countries from achieving those targets: 2 countries would reach them by 2030, 13 between 2030 and 2050, and 13 after 2050.

## Abstract 2

Iván Mejía-Guevara and colleagues predict neonatal and under-5 mortality in sub-Sahara Africa by 2030, with most countries missing Millennium Development Goals for child mortality

## Author summary

**Why was this study done?:**

- Under-5 mortality remains high in sub-Saharan Africa (SSA), and it is declining unevenly across countries and age groups.
- Only a few countries in SSA met the Millennium Development Goals (MDGs) for child mortality reduction.
- A new analysis that explores sources of variation is needed to address concerns on whether the region would be able to reach the Sustainable Development Goals (SDGs).

**What did the researchers do and find?:**

- Using data from full birth histories, we developed a new demographic model to assess age patterns of mortality, fit mortality profiles across age and time, and forecast under-5 mortality.
- We found marked differences in mortality profiles by age and levels across countries over time. The average annual rate of reduction (ARR) for under-5 mortality rates (U5MRs) between 1990 and 2017 was 3.2%, but it varied from 0.1% in Lesotho to 5.2% in Malawi.
- Based on our prediction model, we assessed the achievability of SDGs mortality targets by 2030 (2050 when possible) for 31 SSA countries.
- Countries with a rapid pace of mortality decline would be more likely to meet the sustainable mortality goals (e.g., Rwanda and Tanzania), as opposed to those with a slower pace (e.g., Chad and Nigeria).
- However, the slow pace in neonatal mortality would prevent most countries from reaching the SDGs by 2030, even countries like Ethiopia that have met the MDGs before the 2015 deadline.

**What do these findings mean?:**

- Our analytical approach aims to provide insights for the assessment of sustainable targets of child mortality and to identify the range of epidemiological situations and trajectories.
- The acceleration of neonatal mortality decline is key to advancing the agenda for sustainable development and should be considered in future programmatic assessments.

## Introduction

Under-5 mortality analysis has been critical in evaluating progress toward the Millennium Development Goal 4 (MDG-4) that called for a reduction of under-5 mortality rates (U5MRs) by two-thirds between 1990 and 2015 [1] and more recently toward the Sustainable Development Goal 3 (SDG-3), which aims to reduce neonatal mortality rates (NMRs) to fewer than 12 per 1,000 live births and U5MRs to at least as low 25 per 1,000 births by 2030 [2]. The monitoring of child survival is conducted by the United Nations Inter-agency Group for Child Mortality Estimation (UN IGME) [1], which has adopted a methodology for child mortality estimation [3,4] and regularly updates the resulting mortality levels and trends around the world [4].

The most recent estimates from UN IGME revealed outstanding progress, as the total number of under-5 deaths dropped from 12.6 million in 1990 to 5.4 million in 2017. Yet progress was uneven among and within countries. In particular, 50% of under-5 deaths occur in sub-Saharan Africa (SSA) [4], a region that concentrates 24% of the worldwide under-5 population [5]. In addition, despite the impressive reduction of 58% of under-5 deaths in SSA between 1990 and 2017 (annual rate of reduction [ARR] = 3.2%), the region continues to have the highest under-5 mortality in the world (with 76 deaths per 1,000 live births in 2017). Uneven progress across ages also persists in the region, with nearly 1 million neonatal deaths still occurring every year from 1990 to 2017 (0% decline) and increasing relative to the total under-5 deaths (from 26% in 1990 to 37% in 2017) [4]. This disparate progress in neonatal relative to under-5 mortality decline is observed even in countries that have succeeded in reducing under-5 deaths during the same period; for instance, Ethiopia reached the MDG-4 target 3 years before the 2015 deadline [6,7], yet the share of neonatal to total under-5 deaths increased from 31% in 1990 to 50% in 2017 [4].

Recent evidence reveals uneven trends in the reduction of child mortality rates in low- and middle-income countries (LMICs) across specific population subgroups: by sex [8,9]; by wealth status, with absolute disparities in mortality declining between the poorest and richest households but with persistent relative differences [10,11]; over space, with substantial spatial heterogeneity within countries [12] but some convergence at subnational levels [13]; and for causes of death [14–16]. Methodological work has addressed the inadequacy of traditional life table models applied to child mortality in SSA [17] and small area smoothing with data from sample surveys and demographic surveillance systems [18]. But little attention has been paid to the age distribution of deaths, although recent studies [19–21] do report differences in some causes of death between neonates and older children.

It is well documented that the reduction in child mortality was a key factor for the change in the age distribution of mortality and the increase of life expectancy experienced in the developed world during the 20th century [22,23], as life expectancy is particularly sensitive to mortality reductions at younger ages [24]. With subsequent declines in child mortality over time, we expect that infant deaths in SSA countries will tend to concentrate in the first month of life as postneonatal conditions improve due to the eradication of exogenous mortality causes (e.g., infectious or parasitic diseases, accidental injuries), and then endogenous causes (those associated with genetics, congenital malformations, or injuries connected to birth) would persist [25,26]. This change relates to the classical epidemiological transition model [27], which states that childhood survival (particularly at ages 1–4) benefits the most from the shift of disease patterns and the increase in life expectancy as infectious diseases are progressively displaced by “degenerative and man-made diseases,” although the duration, pace, timing, and determinants have been subject to criticism [28–30].

The main purpose of this study is 3-fold: 1) contribute to filling the gap in modeling fine-grained mortality patterns for under-5 children, 2) the analysis of trends in the age at death distribution for under-5 children, and 3) the forecasting of age patterns and mortality levels by country in SSA.

## Methods

This study follows the guidelines in STrenthening the Reporting of OBservational studies in Epidemiology (STROBE) for reporting observational cross-sectional studies as well as the REporting of studies Conducted using Observational Routinely-collected health Data (RECORD) [31,32]. The analysis is based on information collected from unidentified individuals who provided informed consent prior to the survey interview. Ethical approval for Demographic and Health Surveys (DHSs) was obtained by the ORC Macro Institutional Review Board and by individual review boards within each participating country.

### Data sources

Data are birth histories from 106 DHSs from 31 SSA countries from the period between 1990 and 2017. The DHS program collects health and demographic information mostly for women in reproductive age (15–49 years old) and their children. The survey design is based on a probabilistic, stratified 2-stage sampling plan that defines strata by administrative regions (e.g., states, provinces) and by rural–urban areas within each region. The first-stage sampling frame consists of a list of primary sampling units (PSUs) or enumeration areas (EAs) that covered the entire country and usually were obtained from the latest national census—when available. Each EA was further subdivided into standard size segments of about 100–500 households per segment. In this stage, a sample of predetermined segments is selected randomly with probability proportional to the EA’s measure of size (number of households in EA). In the second stage, DHS survey personnel select households systematically from a list of previously enumerated households in each selected EA segment, and in-person interviews are conducted in selected households to target populations: women aged 15–49, men aged 15–59 (15–54 or 15–49 in some surveys), and children under 5. The number of selected households per EA is variable but ranges from 30 to 40 households/women per rural cluster and from 20 to 25 households/women per urban cluster [33].

### Full birth histories and retrospective mortality data

Full birth history (FBH) data are available for individual women in DHSs, including up to 20 previous births for every eligible woman—usually women 15–49—for which the respondent mother is asked about the date of birth of each of her ever-born children and the age at death if the child has already died [34].

FBH data permits the estimation of death rates for up to 25 years before the survey [34]. We used retrospective information from FBH data, following the statistical guidelines from Pedersen and Liu [35]. We selected the time periods recommended by those authors for the estimation of infant mortality rates (IMRs) and for the countries and survey years considered in their study that matched our sample; for subsequent survey years that were available after their publication, we considered the time interval used in the latest survey included in that study or a 5-year period for countries that were not included. For each country, we then estimated NMRs (death during the first 28 days of life), IMRs (death during the first 12 months of life), and U5MRs (death during the first 59 months of life) retrospectively, starting with year 1990 (or later for some countries) to focus on the period 1990 to 2017 (a complete list of countries/years is in S1 Table).

### Demographic methods

We built our demographic model as follows: 1) we computed conditional life-table age distribution of under-5 deaths from survey data; 2) we adjusted our mortality profiles to match NMRs, IMRs, and U5MRs from UN IGME estimates; and 3) we smoothed out the resulted age mortality profiles and fit and forecast them using a modified version of the Lee–Carter (LC) model. Details for each step are described in what follows.

#### 1) “Conditional” life table age distribution of under-5 deaths

We constructed life-table age distributions of death based on estimated death rates obtained from death reports by households and birth history data from DHSs [34]. We assigned deaths and exposure time across each calendar year on a monthly basis. Estimates of age-specific death rates *m*_*[x]*_ (*[x]* stands for age in months) considered the contributions of children in the survey to the number of events and total time to event [34]. We computed period life table probabilities of dying, *q*_*[x]*_ (probability of dying between month *x* and month *x* + 1), assuming that deaths are distributed uniformly across every single month age range.

$$q_{\left[x\right]}=\frac{\frac{m_{\left[x\right]}}{12}}{\left(1+\frac{m_{\left[x\right]}}{24}\right)}$$ (1)

We derived Eq 1 from a conversion formula (developed by Greville [36] and Chiang [37]) of a set of period age-specific death rates (_*n*_*m*_*x*_, with *n* measured in years) to a set of age-specific probabilities of dying (_*n*_*q*_*x*_), but modified the formula to consider that we measure *n* in months (*n* = 1/12)—details of this conversion are in S1 Text. We estimated NMRs, IMRs, and U5MRs using this methodology and the following formulas [34].

$$NMR=q_{\left[0\right]},$$ (2)

$$IMR=1-\prod_{x=0}^{11}\left(1-q_{\left[x\right]}\right),$$ (3)

$$U5MR=1-\prod_{x=0}^{59}\left(1-q_{\left[x\right]}\right).$$ (4)

#### 2) UN IGME neonatal, infant, and under-5 mortality adjustment

Direct estimates of under-5 deaths based on FBH are prone to measurement errors, as the information is reported directly by living mothers (survivor bias) or due to an upper age limit that is usually considered as an eligibility criterion for surveyed women (truncation bias: eligible women are usually in the age range 15–49) [34,38]. Survivor bias is particularly relevant in countries with extended periods of high HIV prevalence [39].

Because of the lack of high-quality vital registration systems for the countries in our sample, we used mortality estimates from the UN IGME group [1,4] that are designed to mitigate bias and error [3] to validate and adjust our mortality estimates. Specifically, a) we adjusted our raw monthly death rates to match the UN IGME estimates for the neonatal (<1 m), post neonatal (1–11 m), and childhood periods (12–59 m); and b) we used the UN IGME rates to validate the age-adjusted trajectories obtained after smoothing and fitting our model (more details in the following section). For (a), the monthly probabilities of surviving in Eqs 2–4, *p*_[*x*]_ = [1−*q*_[*x*]_], were adjusted proportionally to match UN IGME estimates of NMRs, IMRs, and U5MRs exactly, resulting in 3 measurement errors, *d*_*M*_ = *1-f*_*M*_ *(M* = *nmr*, *pmr*, *cmr)*, in which *f*_*M*_ stands for the adjustment factor applied respectively to _*[*__*1*__*]*_*p*_*[0]*_, _*[*__*11*__*]*_*p*_*[*__*1*__*]*,_ and _*[*__*59*__*]*_*p*_*[*__*12*__*]*_ (_*[a]*_*p*_*[x]*_ is the probability of surviving from the age month *x* to *x* + *a*).

Our direct unadjusted estimates of neonatal mortality differ by as much as 2% from the UN IGME values, in contrast with the postneonatal and child periods that were highly concordant (i.e., with practically no adjustment required) for the majority of year periods. Our unadjusted estimates of neonatal rates were noisy over time, as we expected from our use of retrospective data, and the noise would have been greatly reduced for many countries by a moving average (no average is used in the analyses reported here). In S1 Fig, we show the magnitude of the error (*d*_*M*_) after the adjustment we made to neonatal rates (*f*_*nmr*_) and the much smaller adjustments we made to postneonatal (*f*_*pmr*_) (period between ages 1 and 11 months) and child mortality (*f*_*cmr*_) rates (between ages 12 and 59 months) to exactly fit the UN IGME estimates.

#### 3) Fit and forecasting of mortality trajectories

We used a two-dimensional P-Spline smoothing and generalized linear model (GLM) to smooth our calibrated mortality profiles over ages and years, assuming that the number of deaths at a given rate are Poisson distributed [40]. Following Camarda (2012), [40] the number of deaths and the number of exposures are arranged in *m*_*x*_*n* matrices ***D*** and ***E***, with rows indexed by age and columns indexed by year, respectively—in the one-dimensional case (age dimension), we have a vector of death counts (***d***), exposures (***e***), and mortality hazards (***μ***). The P-Splines consist of a combination of B-Spline basis with roughness penalization (or regularization) on the basis coefficients [41,42], with equally spaced B-Splines used as regression basis and adjusted to our Possion data as follows:

log(E(y))=log(e)+log(μ)=log(e)+Bα, (5)

in which *E*(***y***) = ***e***∙***μ*** (as ***y***~*Poi*(***e***∙***μ***)). Eq 5 represents a GLM with B-Splines as regressors and a log link function of the poisson death counts. With P-Splines, this model is adjusted using an iteratively reweighted least squares (IRWLS) algorithm, but the solution includes a penalization matrix ***P*** that controls the tradeoff between smoothness and model accuracy (tuning of 1 or 2 smoothing parameters is performed during the optimization process). Although the same model specification in Eq 5 and estimation approach can be applied to both one- and two-dimensional data (age and time dimensions), a generalized linear array model (GLAM) [41] is used to adjust the model in two-dimensional settings as the problem may become computationally intractable with large age and time intervals. We used the R package *MortalitySmooth*, which is tailored to model mortality data in one- and two-dimensional settings with P-Splines [40].

A variant of the LC model (Li–Lee–Tuljapurkar [LLT] [43]) is applied to the age mortality profiles after smoothing. In contrast with the standard LC model [44], the modified version is suitable for mortality profiles using data sets that contain multiyear gaps and provides various measurement errors (95% unbiased, narrow, and wide error bounds) if data are available at least for three periods (S1 Text). This characteristic is particularly relevant for this study because our DHS life histories contain year gaps for some countries even after we augmented the periods of analysis using the retrospective information when trying to fill those gaps (as shown in S1 Table). This augmentation did allow us to overcome the requirement of the 3 years of data in the LLT approach [43]. For age *[x]* and year *t*, the LC model that we fit has the form,

$$log\;m_{[x]t}=a_{\left[x\right]}+b_{\left[x\right]}\;k_{t}+e_{[x]t},$$ (6)

in which the first 2 terms on the right are estimated in a singular-value decomposition step, and the last term is an error term whose variance is estimated, as described by Li and colleagues [43]. The term *a*_[*x*]_ represents the average age distribution for each country, *k*_*t*_ tracks mortality changes over time, *b*_[*x*]_ determines how much the age group *[x]* mortality changes with a unit change in *k*_*t*_, and *e*_[*x*]*t*_ represents age-period disturbances not captured by the model. We measured the goodness-of-fit of the LC model as the percentage of the variance explained of the mortality profile *m*_[*x*]*t*_ (after the adjustment to match UN IGME estimates) by the first principal component of the singular-value decomposition (details in S1 Text) [45]. For most countries, the LC model captured more than 90% of the total variation of under-5 mortality (S1 Table).

**Fig 1:**
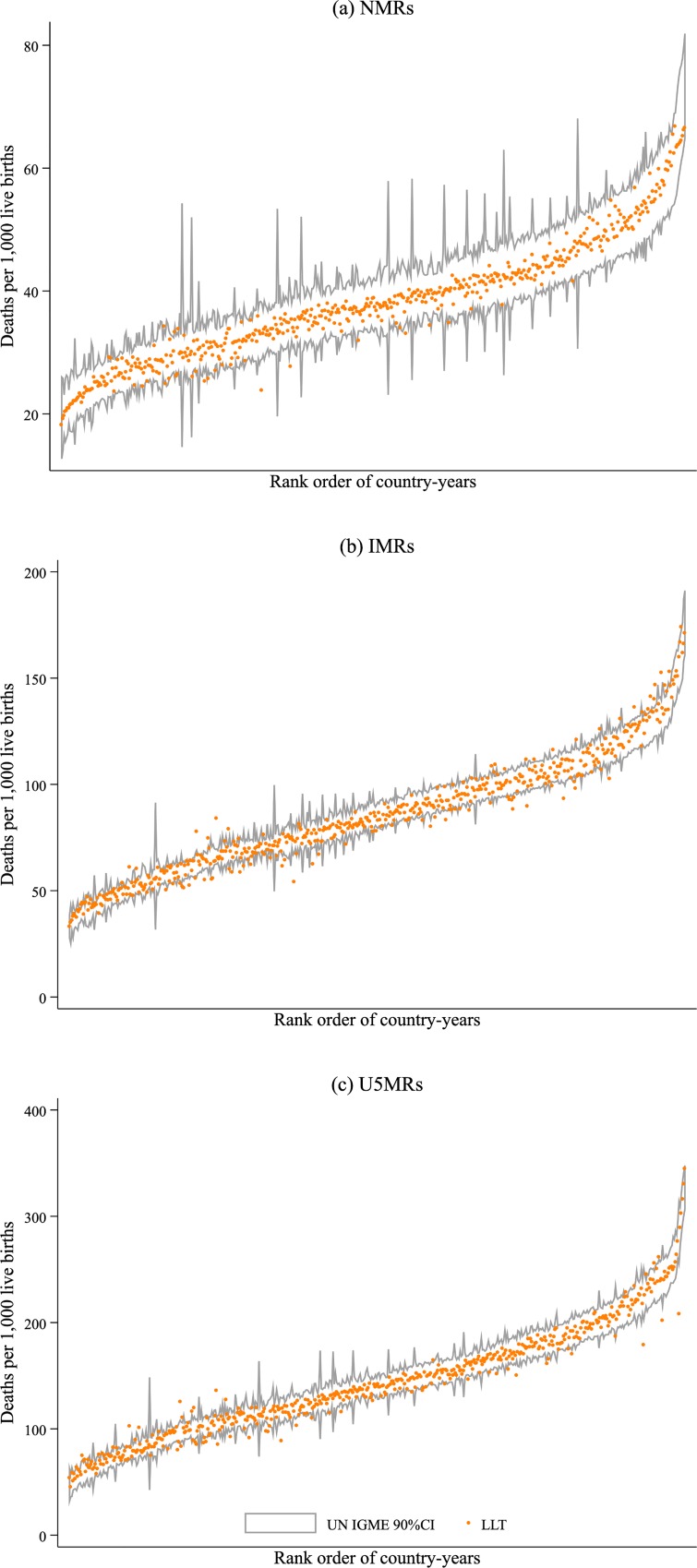
Mortality rates estimated using fitted age profiles from the LLT model and 90% CI from UN IGME estimates for selected year time periods (between 1990 and 2017) in 31 SSA countries. (a) NMRs, (b) IMRs, and (c) U5MRs. Country years represented in the x-axis were sorted on the basis of mortality levels. We retrieved UN IGME estimates from [4]. CI, credible interval; IMR, infant mortality rate; LLT, Li–Lee–Tuljapurkar; NMR, neonatal mortality rate; SSA, sub-Saharan Africa; U5MR, under-5 mortality rate; UN IGME, United Nations Inter-agency Group for Child Mortality Estimation.

The resulting fits were used to generate smoothed-point estimates (the median LC values) of age-specific death rates within the 1990–2017 period and NMR, IMR, and U5MR mortality rates. These estimated rates fell within the 90% credible intervals reported in the latest revision of the UN IGME model (see Fig 1). The dots in that figure represent mortality estimates from our LLT model, and the dark gray lines the limits of credible intervals reported by UN IGME. Around 97%, 84%, and 88% of our neonatal, infant, and under-5 mortality estimates fell within those credible limits, respectively (we dropped Rwanda estimates from the period between 1990–1993, as the resulted mortality rates looked unrealistically high).

## Results

### Trends and forecasts of age-specific mortality patterns

Under-5 mortality has declined for most countries in SSA between 1990 and 2017, but the pace of decline has been uneven and with marked differences across age and countries. That is, U5MR decreased by 3.3% (annual rate of reduction [ARR] 0.1%) (the formula to estimate ARR is in S1 Text) in Lesotho, 59.4% (median) (ARR 3.3%) in Burkina Faso, and 76.4% (ARR 5.2%) in Malawi. By 2017, only 10 countries in our sample had reached/outpaced the MDG-4 target: Ethiopia, Liberia, Malawi, Mozambique, Niger, Rwanda, Senegal, Uganda, the United Republic of Tanzania (Tanzania hereafter), and Zambia. Meanwhile, the decline of NMRs were smaller than U5MRs for all countries, with the lowest, median, and largest declines observed in Lesotho (1.8%, ARR 0.1%), Ghana (42.5%, ARR 2.0%), and Guinea (60.9%, ARR 3.4%), respectively (Table 1). The complete list of countries (31) and years (524) of study are in S1 Table.

**Table 1:** Levels, trends, and forecasts of neonatal, infant, and under-5 mortality.

** **		**Neonatal mortality**										**Infant mortality**										**Under-5 mortality**									
Country		Mortality rates (1) per 1,000 live births and number of deaths (2) in thousands				Decline (%)			ARR (%)			Mortality rates (1) per 1,000 live births and number of deaths (2) in thousands				Decline (%)			ARR (%)			Mortality rates (1) per 1,000 live births and number of deaths (2) in thousands				Decline (%)			ARR (%)		
		1990	2017	2030	2050	1990–2017	2017–2030	2017–2050	1990–2017	2017–2030	2017–2050	1990	2017	2030	2050	1990–2017	2017–2030	2017–2050	1990–2017	2017–2030	2017–2050	1990	2017	2030	2050	1990–2017	2017–2030	2017–2050	1990–2017	2017–2030	2017–2050
Angola	1	54	29	25	15	46.1	12.7	48.6	2.3	1.0	2.0	132	54	44	21	59.2	18.7	60.4	3.3	1.6	2.8	224	81	65	28	63.7	20.4	65.3	3.7	1.7	3.2
	2	35	36									83	65									137	96								
Benin	1	46	33	28	23	29.2	13.0	30.6	1.3	1.1	1.1	107	64	48	34	40.7	24.0	46.5	1.9	2.1	1.9	178	98	73	48	44.7	25.6	50.9	2.2	2.3	2.1
	2	11	13									24	26									39	39								
Burkina Faso	1	46	25	22	15	44.7	12.7	42.2	2.2	1.0	1.6	99	51	43	25	48.3	16.7	50.5	2.4	1.4	2.1	200	81	71	39	59.4	12.2	51.9	3.3	1.0	2.2
	2	19	19									40	37									80	58								
Burundi	1	40	22	15	11	44.8	29.9	50.7	2.2	2.7	2.1	105	43	26	16	59.7	38.9	61.7	3.3	3.7	2.9	175	61	34	20	64.9	44.0	67.4	3.8	4.4	3.3
	2	11	10									28	19									46	27								
Cameroon	1	40	26	25	15	35.4	3.0	41.0	1.6	0.2	1.6	86	55	52	26	35.9	5.6	53.4	1.6	0.4	2.3	139	84	83	38	39.4	0.8	54.6	1.8	0.1	2.4
	2	21	22									44	47									70	71								
Chad	1	52	35	28	20	33.1	18.5	43.2	1.5	1.6	1.7	112	73	58	38	34.5	21.1	48.7	1.6	1.8	2.0	213	123	93	55	42.1	24.8	55.3	2.0	2.2	2.4
	2	16	22									33	46									60	76								
Congo	1	28	19	15	9	30.7	21.6	55.3	1.3	1.9	2.4	59	35	30	16	41.0	12.7	54.3	1.9	1.0	2.3	90	48	46	23	47.2	4.0	52.1	2.3	0.3	2.2
	2	3	3									5	6									8	8								
Cote d'Ivoire	1	48	34	27	20	30.6	18.6	41.2	1.3	1.6	1.6	104	64	55	38	38.3	14.5	40.7	1.8	1.2	1.6	152	89	81	56	41.5	9.2	37.4	2.0	0.7	1.4
	2	26	30									54	56									77	77								
DR Congo	1	42	29	24	18	30.9	17.2	39.2	1.4	1.4	1.5	120	70	52	34	41.5	26.0	51.8	2.0	2.3	2.2	186	91	66	40	51.1	27.5	56.6	2.6	2.4	2.5
	2	67	98									186	233									284	300								
Ethiopia	1	60	29	21	12	51.4	27.1	57.3	2.6	2.4	2.5	120	41	28	15	65.9	32.0	64.6	3.9	2.9	3.1	202	59	37	18	71.0	36.2	69.9	4.5	3.4	3.6
	2	137	95									267	133									439	189								
Gabon	1	31	22	18	14	30.6	17.3	35.9	1.3	1.4	1.3	60	35	26	19	41.7	24.6	46.5	2.0	2.1	1.9	92	48	42	30	47.7	13.1	37.8	2.4	1.1	1.4
	2	1	1									2	2									3	3								
Gambia	1	50	28	21	13	44.7	25.5	52.4	2.2	2.2	2.2	82	41	30	18	49.8	28.5	56.8	2.5	2.5	2.5	170	64	41	22	62.6	35.1	64.8	3.6	3.3	3.1
	2	2	2									3	3									7	5								
Ghana	1	42	24	19	16	42.5	21.5	35.8	2.0	1.8	1.3	79	36	26	20	55.0	27.1	43.6	2.9	2.4	1.7	127	49	34	25	61.0	31.8	49.5	3.4	2.9	2.1
	2	24	21									44	31									70	43								
Guinea	1	62	24	13	7	60.9	47.9	70.6	3.4	4.9	3.6	139	56	29	16	59.3	48.8	70.8	3.3	5.0	3.7	235	86	47	27	63.5	45.1	68.9	3.7	4.5	3.5
	2	18	11									38	25									63	38								
Kenya	1	30	21	16	12	29.6	22.3	40.6	1.3	1.9	1.6	66	34	24	16	49.0	29.6	52.1	2.5	2.7	2.2	104	46	32	20	56.0	29.7	55.4	3.0	2.7	2.4
	2	29	32									64	51									101	69								
Lesotho	1	39	38	40	39	1.8	−6.7	−3.1	0.1	−0.5	−0.1	71	67	85	91	6.6	−28.5	−37.1	0.3	−1.9	−1.0	89	86	124	134	3.3	−44.0	−56.0	0.1	−2.8	−1.4
	2	2	2									4	4									5	5								
Liberia	1	58	25	15	7	57.0	39.4	73.7	3.1	3.8	4.0	174	56	27	9	67.8	51.7	83.4	4.1	5.4	5.3	261	75	37	12	71.3	50.4	84.4	4.5	5.3	5.5
	2	6	4									16	9									25	12								
Malawi	1	50	23	15	8	54.5	35.4	64.3	2.9	3.3	3.1	138	39	23	11	72.1	39.6	72.0	4.6	3.8	3.8	235	55	35	15	76.4	37.3	73.5	5.2	3.5	3.9
	2	22	15									60	26									99	37								
Mali	1	73	35	26	16	51.4	25.8	53.7	2.6	2.3	2.3	130	66	45	26	49.4	31.2	59.9	2.5	2.8	2.7	254	106	75	41	58.3	29.7	61.6	3.2	2.7	2.9
	2	30	28									53	50									101	80								
Mozambique	1	58	27	17	9	53.7	36.8	66.4	2.8	3.5	3.2	159	53	30	13	66.6	43.4	74.8	4.0	4.3	4.1	240	72	42	18	69.8	41.8	75.6	4.3	4.1	4.2
	2	36	31									96	60									144	81								
Namibia	1	28	18	12	8	36.8	29.2	52.8	1.7	2.6	2.2	49	32	38	34	35.1	−19.2	−8.4	1.6	−1.4	−0.2	73	44	62	58	39.2	−39.9	−30.7	1.8	−2.6	−0.8
	2	1	1									3	2									4	3								
Niger	1	54	26	18	10	52.1	30.6	60.9	2.7	2.8	2.8	132	48	30	15	63.4	36.9	69.6	3.7	3.5	3.5	327	85	47	19	74.1	44.3	77.5	4.9	4.4	4.4
	2	24	27									57	49									136	82								
Nigeria	1	50	33	26	18	34.6	20.1	43.8	1.6	1.7	1.7	126	65	48	30	48.6	24.9	53.3	2.4	2.2	2.3	212	100	73	42	52.7	27.2	57.6	2.7	2.4	2.6
	2	212	241									514	466									857	714								
Rwanda	1	40	16	10	4	59.1	39.0	74.5	3.3	3.7	4.1	93	29	16	5	68.8	45.1	81.1	4.2	4.5	4.9	151	38	22	7	74.9	42.4	82.7	5.0	4.2	5.2
	2	13	6									29	11									48	14								
Senegal	1	40	21	15	10	48.5	26.9	51.5	2.4	2.4	2.2	71	33	23	15	54.1	29.5	55.4	2.8	2.7	2.4	139	45	29	17	67.4	37.0	63.5	4.1	3.5	3.0
	2	13	11									23	18									44	25								
Sierra Leone	1	53	34	34	21	37.0	−1.1	37.1	1.7	−0.1	1.4	155	82	82	43	47.4	−0.1	47.9	2.4	0.0	2.0	262	111	115	54	57.8	−4.4	51.2	3.1	−0.3	2.2
	2	10	9									30	21									50	28								
Togo	1	43	25	18	12	41.2	30.1	53.0	1.9	2.7	2.3	90	49	31	20	45.4	36.1	59.8	2.2	3.4	2.7	146	73	46	27	50.1	36.7	62.3	2.5	3.5	2.9
	2	7	7									14	13									23	19								
Uganda	1	40	20	16	11	49.6	23.1	46.9	2.5	2.0	1.9	107	35	22	13	67.0	37.1	63.3	4.0	3.5	3.0	181	49	28	15	73.0	42.6	70.0	4.7	4.2	3.6
	2	35	36									90	62									148	85								
Tanzania	1	38	21	15	10	44.2	30.8	52.5	2.1	2.8	2.2	100	38	21	13	61.8	44.8	67.0	3.5	4.5	3.3	166	54	28	15	67.4	48.7	72.0	4.1	5.0	3.8
	2	43	46									110	82									178	114								
Zambia	1	37	22	18	10	40.6	20.9	55.1	1.9	1.8	2.4	111	42	28	12	62.4	32.9	69.9	3.6	3.0	3.6	185	60	37	14	67.6	37.6	75.9	4.1	3.6	4.2
	2	14	14									39	26									64	38								
Zimbabwe	1	26	22	21	19	12.2	7.5	13.7	0.5	0.6	0.4	51	37	38	35	28.1	−4.9	4.4	1.2	−0.4	0.1	78	50	59	52	35.1	−16.4	−4.1	1.6	−1.2	−0.1
	2	10	12									19	19									29	27								

**Abbreviation:** ARR, annual rate of reduction; LLT, Li–Lee–Tuljapurkar; UN IGME, United Nations Inter-agency Group for Child Mortality Estimation.

Mortality rates and number of deaths from 1990 and 2017 are official estimates from UN IGME [4], while values from 2030 and 2050 were estimated using the LLT model.

**Fig 2:**
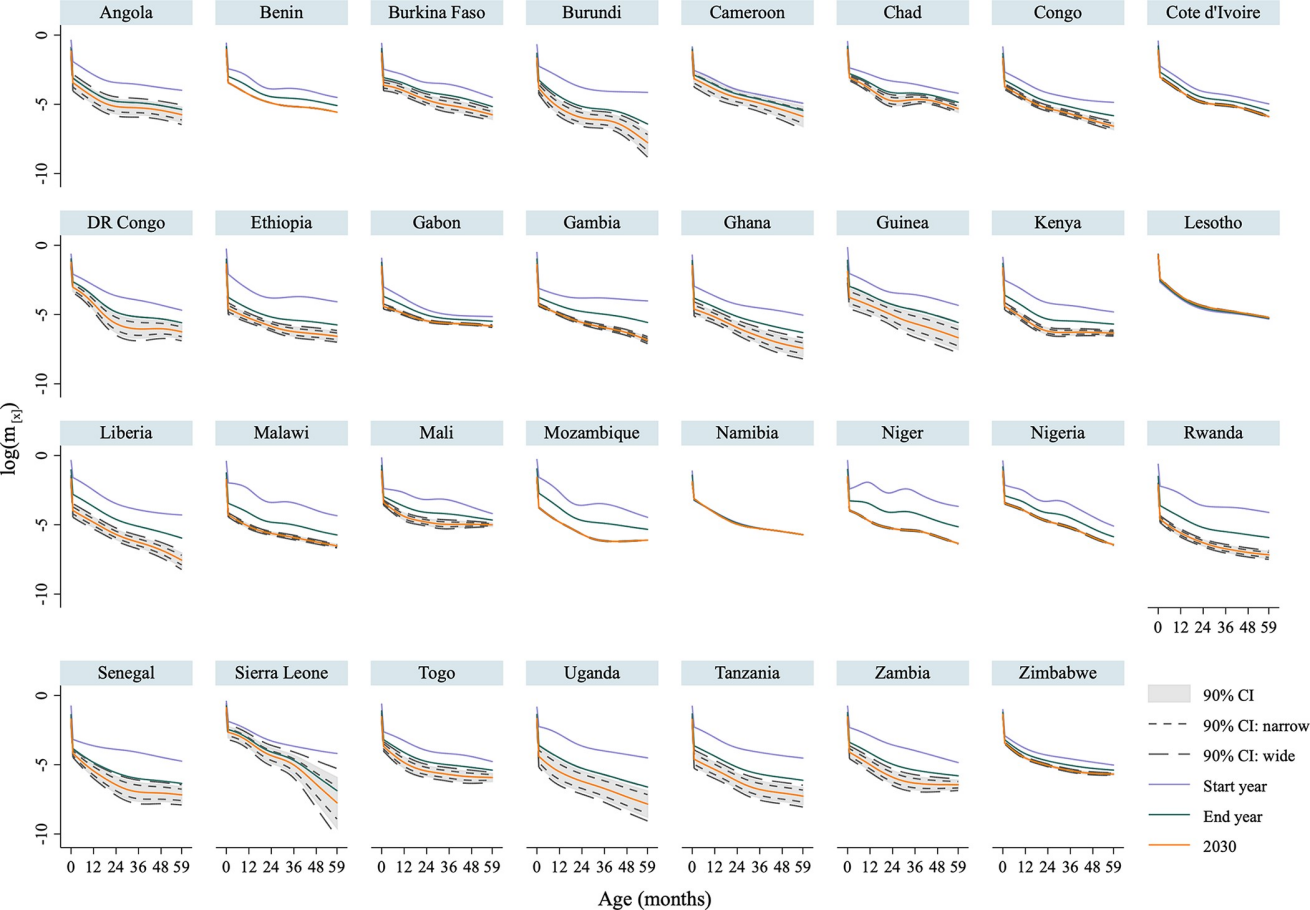
LLT fit and prediction of age patterns of death rates for under-5 children in 31 SSA countries. Author’s estimates using the LLT model [43] with data from the DHS program. CI, credible interval; DHS, Demographic and Health Survey; LLT, Li–Lee–Tuljapurkar; SSA, sub-Saharan Africa.

We used our LLT model to fit and forecast U5MRs for every country. The forecasts of trajectories were for the years 2030 and 2050 (predictions for Lesotho were precluded by the poor quality of data and great uncertainty in the estimates and uncertainties). Most countries display a monotonic decline of death rates at every age, but there are notable differences in the age patterns over time. Death rates by age for all countries are in Fig 2, including fitted (between 1990 and 2017) and predicted (2030) age-specific mortality trajectories. For instance, Chad, Nigeria, Rwanda, and Tanzania are representative countries with different trajectories of mortality reduction. Chad and Nigeria are countries with low ARR (below 3.2%, the ARR of SSA between 1990 and 2017 [4]), whereas Rwanda and Tanzania represent countries with high ARR (above 3.2%). For Chad, mortality fell most rapidly at ages between 1 and 3 years, leading to a decidedly uneven pattern (by age) in 2015 and persisting to 2030. However, in Nigeria, although death rates during the second year of life decreased more rapidly in the initial period, mortality at all ages eventually declined at similar proportional speeds, and the mortality profile remains linear with age to 2030. Our prediction model for both countries indicates an improvement in the mortality levels at all ages by 2030 but with more uncertainty in Chad. Mortality patterns in Rwanda and Tanzania are similar; the transition from high to low mortality across the under-5 period starts with a sharp decline in infant (2–11 m) and child (12–59 m) mortality, followed by a less rapid decline in neonatal (<1 m) mortality. In both countries, however, the mortality profile is steepest during the infant period compared to the child period across time. Our model also predicts significant declines of mortality by 2030 at all ages, the mortality curve in Rwanda becoming increasingly rectangular, i.e., concentrating near birth and then falling sharply and rapidly with age.

Our forecasting model revealed that countries that experienced a low pace of under-5 mortality decline in the past would most likely fall short in achieving the SDG-3 target for under-5 mortality by 2030, in contrast with those experiencing more substantial or accelerated reductions. However, the lower pace of neonatal mortality decline observed in the region during our period of investigation may prevent most countries from reaching both the neonatal and under-5 SDG-3 target by 2030 (fewer than 12 or at least as low 25 deaths per 1,000 live births for neonatal and under-5 targets, respectively), including countries with a higher pace of under-5 mortality reduction. For example, the ARR for neonatal and under-5 mortality in Nigeria between 1990 and 2017 of 1.6 and 2.7, respectively, would remain the same or decrease between 2017 and 2030 (and 2050), and that would prevent the country from achieving the SDG-3 targets—neither in 2030 nor in 2050 (Table 1, S2 Fig). In contrast, the ARR of neonatal and under-5 mortality in Guinea and Rwanda would increase or remain high (above 3.2%) after 2017, and as a consequence, these countries would likely reach those targets (Table 1, S2 Fig). Rwanda and Tanzania, 2 countries that have achieved MDG-4 [46], would likely meet the SDGs of neonatal and under-5 mortality reduction by 2030, according to our predictions. However, although Ethiopia has also reached MDG-4 since 2013 and experienced reductions of 51% and 71% in neonatal and U5MRs between 1990 and 2017, respectively, the country would likely fall short of SDG-3 because of its slower rate of neonatal decline—the ARR was 2.6% between 1990 and 2017, and it is expected to remain at that level (approximately 2.5%) afterwards (Table 1).

**Fig 3:**
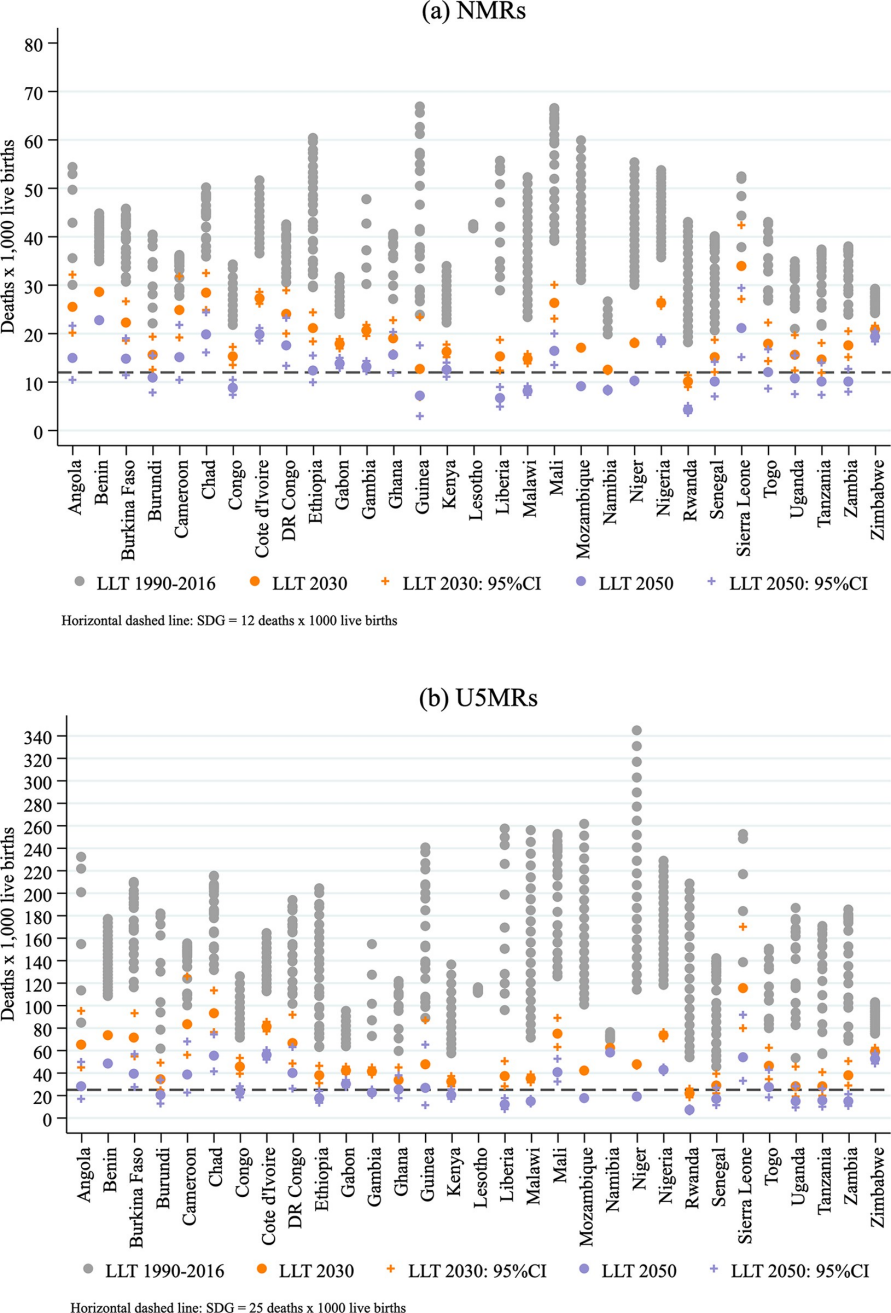
Forecasts of NMRs and U5MRs by 2030 and 2050 based on the LLT model and assessment of SDG-3 targets in 31 SSA countries. (a) NMRs and (b) U5MRs. Predictions for Lesotho were precluded by the poor quality of data and great uncertainty in the estimates and uncertainties. We report unbiased error bounds for our prediction models for 2030 and 2050. LLT, Li–Lee–Tuljapurkar; NMR, neonatal mortality rate; SDG-3, Sustainability Development Goal 3; SSA, sub-Saharan Africa; U5MR, under-5 mortality rate.

In summary, we predict that only five countries are on track to achieve the under-5 mortality target of SDG-3 by 2030 (Kenya, Rwanda, Senegal, Tanzania, and Uganda) (Fig 3), but only Rwanda and Tanzania would also meet the neonatal mortality target by 2030. If the observed pace of mortality reduction continues, and considering the uncertainty predicted by our model (based on our estimated unbiased error bounds), we predict that only 13 countries would achieve the neonatal and under-5 goals between 2030 and 2050 (Angola, Burundi, Cameroon, Congo, Ethiopia, Ghana, Kenya, Liberia, Malawi, Mozambique, Niger, Togo, and Zambia), and 13 more would make it after 2050 (Benin, Burkina Faso, Chad, Cote d’Ivoire, Democratic Republic of the Congo [DR Congo], Gabon, Gambia, Lesotho, Mali, Namibia, Nigeria, Sierra Leone, and Zimbabwe) (Fig 3).

## Discussion

This paper describes 3 novel findings. First, we advanced the modeling of age patterns of under-5 mortality for detailed age groups using FBH data from SSA, providing important information on under-5 mortality patterns and representing a step forward in the analysis of changes in age patterns of mortality across time and by country. We used the latest refinements in the estimation of child mortality based on full birth histories from survey data and adjusted and validated our rates using official estimates of U5MRs that are derived from a robust model [1,3]. Second, we made probabilistic projections of age patterns of mortality by 2030 (and where possible to 2050) in order to assess progress toward the SDGs of child mortality reduction. In making that assessment, our use of probabilistic methods allowed us to account for different degrees of uncertainty. Our predictions are consistent with estimates from UN IGME, as we found discrepancies for only four countries in the timing where SDG-3 would be achieved. Third, we predict that Kenya, Rwanda, Senegal, Tanzania, and Uganda are on track to achieve SDG-3 for under-5 mortality reduction, but only Rwanda and Tanzania would meet the neonatal target as well, and 13 countries would achieve both targets only after 2050.

This study is in line with previous findings of under-5 mortality reduction in SSA [4] but goes further by showing the reductions in age-specific death rates on a monthly basis for the majority of countries in the region. It also identified heterogeneities in the trends and age patterns of mortality decline across countries, with important lags in most countries that would prevent them to reach the SDG targets. Although a complete assessment on the specific determinants of mortality decline across ages requires further research, recent evidence suggests that more rapid rates of decline of communicable, maternal, neonatal, and nutritional diseases than noncommunicable diseases have had different impacts across ages in early mortality [16]. However, heterogeneous effects of the observed transition, between and within regions, can be attributable to differences in the successful introduction and implementation of programs and policies, social determinants, and persistent causes of mortality (preventable or from regions/countries with conflicts/civil unrest/high HIV prevalence) [12,46,16,11,47]. Further progress toward the achievement of SDGs in SSA would require accelerated rates of decline of noncommunicable diseases and to attend the underlying determinants of heterogenous effects across and within countries.

Recent studies for countries that achieved the MDG-4 targets revealed important insights into the determinants of change, coverage, intervention, and implementation of policies that have succeeded in specific contexts. Ethiopia has developed multisectoral policy platforms that integrate child survival and specific health goals within macrolevel policies and programs [48,6,7]. Niger has developed policies intended to increase access to child health services, the use of mass campaigns, and programming for nutrition [49]. Tanzania put high political priority on child survival, with consistent increases in funding, and focused on the implementation of high-impact interventions at lower levels of the health system, although to the detriment of mothers and neonates [50]. After the Rwandan genocide in 1994 that led to the death of more than 1 million people and the devastation of the health system [51], the country embarked on ambitious programs to provide equitable health services that resulted in the improvement of health equity and child survival. The rebuilding of the health system included notions of ready access, accountability, and solidarity, as well as the implementation and scale-up of community-based health insurance and performance-based financing systems [52]. However, the progress of maternal and neonatal outcomes has been slower in general, mainly due to low coverage to intrapartum interventions, lower political commitment, less financing, health system constraints, and low or unequal rates of health facility delivery [46,53]. These experiences should guide policies and interventions for the majority of countries expected to fall short in achieving the SDG-3 targets but particularly for those that would not meet them before 2050, as predicted by our model.

Disparities in socioeconomic and regional factors also have the potential to generate or preserve heterogeneities in mortality risks across under-5 age groups, as interventions, accessibility, timing, and quality of health services may generate inequalities in the exposure to mortality risk. Recent evidence suggests that absolute disparities in mortality reduction between the poorest and richest households have declined in SSA [11,54], but persistent relative differences remain [11]. Similarly, the urban advantage in under-5 mortality over time prevails in SSA, and the growth of urban populations predicts the magnitude of under-5 disparities [55]. Regional differences in mortality risk can be observed in both countries with slow [56] or rapid gains in child survival or that have met the MDGs [48–50,55,57,58]. In Nigeria, for example, wide variations in under-5 mortality are observed across states over time, with higher levels in northern regions. Subnational health trends, further stratified by socioeconomic status, region, and age, can improve our understanding of the health challenges in the region and should inform further efforts to reduce the mortality risk and the successful achievement of SDG-3.

Despite the accelerated progress in the reduction of under-5 deaths observed in some countries, the mechanisms and underlying factors leading to improvements in child survival, which could undermine or decelerate progress in mortality reductions for specific age groups, require further examination. For instance, the global mortality rank in pneumonia and diarrhea deaths in under-5 children, the 2 diseases responsible for about 25% of all of the deaths that occurred in under-5 children in 2015, reveals that 72% of the global burden of pneumonia and diarrhea child deaths occurred in just 15 countries [59]. Seven countries in our study sample (Angola, Chad, DR Congo, Ethiopia, Niger, Nigeria, and Tanzania) are among them, but only Chad and Nigeria have registered ARR below 3.2% (Table 1). That is, countries that have achieved significant progress in the reduction of mortality in the past (i.e., Ethiopia, Niger, and Tanzania) still face significant challenges with the potential to affect the age distribution of deaths. In addition, although previous studies did not attribute the emergence of HIV as the leading cause of modifying preexisting patterns of under-5 mortality in some SSA regions [17], our findings indicate that countries largely affected by the HIV/AIDS epidemy showed signs of poor data quality and unreliable fit and predictions (Lesotho, Namibia, and Zimbabwe) but also substantive progress in child mortality reduction during the previous 2 decades (Malawi).

Our model differs in the way to approach mortality estimation from 2 leading models in the literature, that we refer here as the UN IGME model [3] and the Institute for Health Metrics and Evaluation (IHME) model [60,61]. First, our method only uses complete birth histories as the main source of information, as opposed to UN IGME and IHME that additionally consider further data sources, including direct recall of household deaths, summary birth histories, sample surveillance (only IHME), and Vital Registration. Second, both models apply a data synthesis approach after a detailed data quality assessment; however, they differ in their data and modeling assumptions: UN IGME is based on a Bayesian penalized B-Spline regression (and multilevel) model, and IHME is based on a Gaussian process regression (GPR) model that incorporates covariates [62]. Third, our method generates detailed age-specific death rates for both sexes combined, whereas UN IGME and IHME are age–sex models that break down into ages 0 and 1–4 (UN IGME) or yearly birth cohorts divided into 52 birth-week cohorts and followed to age 5 (IHME) [62]. Although there is room for improvement in our model by incorporating further data sources and disaggregation by sex, our methodological approach is simpler and our assessment of SDG-3 progress is in line with estimates from both UN IGME and IHME in the majority of countries in our sample. That is, we identified only 4 countries with discrepancies in both neonatal and under-5 mortality predictions from UN IGME: Cameroon, Togo, Tanzania, and Zimbabwe. In Cameroon, Togo, and Tanzania, the difference is that we predict an early transition toward the SDG-3 targets (S2 Fig). Further comparisons with predictions from the IHME model revealed that our estimates (and those from UN IGME) are less optimistic in general, although we found an overlap in 26 and 23 countries (out of 31) for neonatal and under-5 estimates, respectively (i.e., IHME predictions are within our estimated error bounds or ours within IHME error bounds). Furthermore, our point estimates for NMRs and U5MRs coincide almost exactly with those of the IHME model for 11 and 12 countries, respectively (S3 Fig).

Our study builds on previous research that examines patterns of mortality as a way to understand the sources of error or true epidemiological patterns that are not captured by model life table approaches for SSA [17,18,63–65] and stress the importance of using new methodological approaches and complementary sources of data [18]. In addition, we complement previous studies that analyze trends and prediction of mortality rates worldwide [4,14,19,20].

To the best of our knowledge, our study is the first to construct under-5 mortality patterns from narrow-age groups using an LLT model for the assessment of trends and prediction of under-5 mortality in SSA with uncertainty. Specifically, we made predictions of mortality rates by 2030 for the assessment of the SDG-3 targets and by 2050 to evaluate which countries in our predictions would meet the SDG-3 targets by then if they fall short to do so by 2030. Our mortality patterns provided evidence of an acceleration of mortality decline and substantive changes in age mortality patterns in countries with higher rates of child survival. In particular, we observed in certain countries that the distribution of deaths would follow a pattern that is becoming increasingly rectangular, having an increasingly flat down and sharp upslope. We refer to this phenomenon as the “early rectangularization” of the under-5 mortality curve, a phenomenon similar but very distinct in nature from the hypothesized rectangularization at old ages, extensively studied in the literature for older populations [66–68]. Further analyses of compression and convergence of early mortality would provide more insights about this phenomenon.

This study has a number of limitations. First, the gold standard for the analysis of mortality in more developed countries relies on the existence of high quality vital registration systems, but those systems are inexistent or deficient in the countries included in our study [69]. Second, this study relies on self-reported information from life histories available in nationally representative surveys, which are subject to several sources of error; estimates for specific countries (i.e., Lesotho and Malawi) may be affected by these data limitations across time, and these need to be taken with caution. Third, because of the nature of the survey data, we are not able to make a detailed assessment of the underlying causes of mortality reduction across the under-5 period.

## Conclusion

This study contributes to the development of detailed age patterns of mortality for under-5 children and stresses their importance in the monitoring of child survival of specific age groups to identify distinct patterns of mortality decline at early ages in most countries of SSA. Our estimates and forecasts relied on a robust LLT model that was suitable for our data with year gaps, providing different degrees of uncertainty and capturing most of the variation of under-5 mortality in the SSA region. Its accuracy could be refined if further reliable sources of information become available, such as the development of new vital registration systems. It should also be considered in the design and scale-up of targeted interventions intended to accelerate progress toward achieving the SDG-3 targets for child mortality reduction. Future research should explore a detailed assessment of age inequality in early mortality, compression, and convergence, as well as the true relationships between age patterns of mortality and epidemiological trajectories.

## Supporting information

S1 RECORD STROBE and RECORD checklists. RECORD, REporting of studies Conducted using Observational Routinely-collected health Data; STROBE, STrenthening the Reporting of OBservational studies in Epidemiology.
(DOCX)

S1 Text Supplementary methods. (PDF)

S1 Table Year of analysis; ARR_NMR_, ARR_IMR_, and ARR_U5MR_; and percentage of VE before (VE_DHS_) and after (VE_UNIGME_) adjusting DHS data to match UN IGME estimates. A brief note with formula to estimate VE is in S1 Text. Highlighted years correspond to the DHS type and year available for this analysis. For Liberia, the latest survey year was not used for analysis but to construct retrospective years for analysis. ARR, annual rate of reduction; DHS, Demographic and Health Survey; IMR, infant mortality rate; NMR, neonatal mortality rate; U5MR, under-5 mortality rate; UN IGME, United Nations Inter-agency Group for Child Mortality Estimation; VE, variance explained.
(PDF)

S1 Fig Difference in survival rates that resulted after adjusting DHS data to match UN IGME estimates for the neonatal (*d_NMR_*), post neonatal (*d_PMR_*), and child (*d_CMR_*) period from 31 SSA countries by year. Authors’ estimates using data from the DHS Program. PMR is the probability of dying between 1 month (28 days) and 11 months of age, expressed per 1,000 live births, and the CMR is the probability of dying between 1 and 4 years of age, expressed per 1,000 children age 1 [4]. CMR, child mortality rate; DHS, Demographic and Health Survey; PMR, postneonatal mortality rate; SSA, sub-Saharan Africa; UN IGME, United Nations Inter-agency Group for Child Mortality Estimation.
(TIFF)

S2 Fig Assessment of the SDG-3 targets for NMRs and U5MRs by 2030 and 2050 based on estimates from the LLT model and from UN IGME for 31 countries from SSA. In the LLT, we report wide error bounds for our prediction models for 2030 and 2050. We retrieved UN IGME estimates online from http://data.unicef.org/topic/child-survival/child-survival-sdgs/#. LLT, Li–Lee–Tuljapurkar; NMR, neonatal mortality rate; SDG-3, Sustainability Goal 3; SSA, sub-Saharan Africa; U5MR, under-5 mortality rate; UN IGME, United Nations Inter-agency Group for Child Mortality Estimation.
(TIF)

S3 Fig Assessment of the SDG-3 targets for NMRs and U5MRs by 2030 based on estimates from the LLT model and from the IHME for 31 countries from SSA. (a) NMRs and (b) U5MRs. In LLT, predictions for Lesotho were precluded by the poor quality of data and great uncertainty in the estimates and uncertainties, and we report unbiased error bounds for our prediction models for 2030. We retrieved IHME estimates online from https://vizhub.healthdata.org/sdg/. IHME, Institute for Health Metrics and Evaluation; LLT, Li–Lee–Tuljapurkar; SDG-3, Sustainability Development Goal 3; SSA, sub-Saharan Africa; U5MR, under-5 mortality rate.
(TIF)

## Abbreviations

ARR: annual rate of reduction
CI: credible interval
CMR: child mortality rate
DHS: Demographic and Health Survey
EA: enumeration area; FBH, full birth history
GLAM: generalized linear array model
GLM: generalized linear model
GPR: Gaussian process regression
IHME: Institute for Health Metrics and Evaluation
IMR: infant mortality rate
IRWLS: iteratively reweighted least squares
LC: Lee–Carter
LLT: Li–Lee–Tuljapurkar
LMIC: low- and middle-income country
MDG: Millenium Development Goal
NMR: neonatal mortality rate
PHS: Center for Population Health Sciences
PMR: postneonatal mortality rate
PSU: primary sampling unit
RECORD: REporting of studies Conducted using Observational Routinely-collected health Data
SDG: Sustainable Development Goal
SSA: sub-Saharan Africa
STROBE: STrenthening the Reporting of OBservational studies in Epidemiology
U5MR: under-5 mortality rate
UN IGME: United Nations Inter-agency Group for Child Mortality Estimation
