# Supplementary material for: Age distribution, trends, and forecasts of under-5 mortality in 31 sub-Saharan African countries: A modeling study
Source: PLoS Med. 2019 Mar 12;16(3):e1002757. doi: 10.1371/journal.pmed.1002757 (PMC6413894; doi:10.1371/journal.pmed.1002757)
Supplement: S1 Text — (PDF) [file pmed.1002757.s002.pdf]

Age distribution, trends, and forecasts of under-5 mortality in 31 sub-Saharan African countries: A modeling study

Iván Mejía-Guevara[1,2,*], Wenyun Zuo[1], Eran Bendavid[3], Nan Li[4], Shripad Tuljapurkar[1]

1 Department of Biology, Stanford University, Stanford, California, United States of America,
2 Stanford Center for Population Health Sciences, Stanford University School of Medicine, Stanford, California, United States of America,
3 Primary Care and Population Health, Stanford University School of Medicine, Stanford, California, United States of America,
4 United Nations Population Division, New York, New York, United States of America

* imejia@stanford.edu

Supplementary methods

"Conditional" life-table age distribution of under-5 death

To estimate period life-table probabilities of dying, we build on Greville [1] and Chiang [2], who derived the following formula for the conversion of death rates between ages x and $x+n$ (${}_nm_x$, n represents years) and the probability of dying during the same interval (${}_nq_x$):

$$ {}_nq_x = \frac{n \cdot {}_nm_x}{1 + (n - {}_na_x)_n m_x}, \tag{S1}$$

where ${}_na_x$ stands for the average person-years lived between x and $x+n$ by those dying in that interval. We use this formula to estimate probabilities of dying in month intervals by setting $n = 1/12$. We also assume that deaths are distributed uniformly across the month age range; that is, on average, persons dying in the month interval do so half-way through the interval and then ${}_na_{[x]} = (1/12) * (1/2) = 1/24$. After these assumptions, we get:

$$ q_{[x]} = \frac{\frac{m_{[x]}}{12}}{1 + \frac{m_{[x]}}{24}}. \tag{S2}$$

Notice that we modified the notation to indicate that age is measured in month intervals: now $[x]$ stands for age in months, and $m_{[x]}$ and $q_{[x]}$ represent monthly death rates and probabilities of dying, respectively.

Two-dimensional P-Spline smoothing

We used a two-dimensional P-Spline smoothing and generalized linear model (GLM) to smooth our calibrated mortality profiles over ages and years, assuming that the number of deaths at a given rate are Poisson-distributed [3]. That is, if $\boldsymbol{D}_{[x]t}$, $\boldsymbol{E}_{[x]t}$, $\boldsymbol{\mu}_{[x]t}$ represent the number of deaths, the exposures and the mortality hazard at age $[x]$ at time t, respectively, then $\boldsymbol{D}_{[x]t} \sim Poi(\boldsymbol{E}_{[x]t} \cdot \boldsymbol{\mu}_{[x]t})$. Following Camarda (2012) [3], the number of deaths and the number of exposures are arranged in $m \times n$ matrices $\boldsymbol{D}$ and

$\boldsymbol{E}$, with rows indexed by age and columns indexed by year, respectively – in the one dimensional case (age dimension), we have a vector of death counts ($\boldsymbol{d}$), exposures ($\boldsymbol{e}$), and mortality hazards ($\boldsymbol{\mu}$). The P-Splines consist of a combination of B-Spline basis with roughness penalization (or regularization) on the basis coefficients [4, 5], with equally-space B-Splines used as regression basis and adjusted to our Poisson data as follows:

$$\log\left(E(\boldsymbol{y})\right) = \log\left(\boldsymbol{e}\right) + \log\left(\boldsymbol{\mu}\right) = \log\left(\boldsymbol{e}\right) + \boldsymbol{B}\boldsymbol{\alpha}, \tag{S3}$$

in which $E(\boldsymbol{y}) = \boldsymbol{e}\cdot\boldsymbol{\mu}$ (as $\boldsymbol{y} \sim Poi(\boldsymbol{e}\cdot\boldsymbol{\mu})$). Eq (S3) represents a GLM with B-Splines as regressors and a log link function of the poisson death counts. With P-Splines, this model is adjusted using an iteratively reweighted least squares (IRWLS) algorithm, but the solution includes a penalization matrix $\boldsymbol{P}$ that controls the tradeoff between smoothness and model accuracy (tuning of 1 or 2 smoothing parameters is performed during the optimization process).

This linear prediction model is adjusted using an IRWLS algorithm, which yields the following estimates for $\boldsymbol{\alpha}$:

$$(\boldsymbol{B}^T\widetilde{\boldsymbol{W}}\boldsymbol{B} + \boldsymbol{P})\tilde{\boldsymbol{\alpha}} = \boldsymbol{B}^T\widetilde{\boldsymbol{W}}\tilde{\boldsymbol{z}}, \tag{S4}$$

where $\tilde{\boldsymbol{z}} = \frac{\boldsymbol{y}-\boldsymbol{e}\tilde{\boldsymbol{\mu}}}{\boldsymbol{e}\tilde{\boldsymbol{\mu}}} + \boldsymbol{B}\tilde{\boldsymbol{\alpha}}$ is a working dependent variable with $\tilde{\boldsymbol{\mu}}$ and $\tilde{\boldsymbol{\alpha}}$ denote current approximations to the solution, and $\widetilde{\boldsymbol{W}}$ is a diagonal matrix of weights ($\widetilde{\boldsymbol{W}} = diag(\boldsymbol{e}\tilde{\boldsymbol{\mu}})$) [4]. The term $\boldsymbol{P}$ in Eq (S4) is defined as $\boldsymbol{P} = \lambda\boldsymbol{D}_k^T\boldsymbol{D}_k$ and represents the main characteristic of the P-Splines model, which is an extension of the standard solution for fitting GLM.

Although the same model specification in Eq (S3) and estimation approach can be applied to both one- and two-dimensional data (age and time dimensions), a generalized linear array model (GLAM) [4] is used to adjust the model in two-dimensional settings as the problem may become computationally intractable with large age and time intervals. More details of this procedure are reported by Camarda [3], who developed the R package ***MortalitySmooth***, and specifically tailored to model mortality data in one- and two-dimensional settings with P-Splines.

Lee-Carter forecast in populations with limited data

For age $[x]$ and year t, the Lee-Carter (LC) model that we fit has the form,

$$\log m_{[x]t} = a_{[x]} + b_{[x]}k_t + e_{[x]t}, \tag{S5}$$

where the first 2 terms on the right are estimated in a singular-value decomposition step, and the last term is an error term whose variance is estimated as described by Li and colleagues [6]. The term $a_{[x]}$ represents the age distribution of the latest observed month for each country, k_t tracks mortality changes over time, $b_{[x]}$ determines how much the age group $[x]$ mortality changes with a unit change in k_t, and $e_{[x]t}$ represents age-period disturbances not captured by the model. We measured the goodness-of-fit of the LC model as the percentage of the variance explained of the mortality profile ($m_{[x]t}$ – after the adjustment to match UN IGME estimates) by the first principal component of the singular-value decomposition, which we compute as:

$$VE = 1 - \frac{\sum_t \parallel \varepsilon_t^{(k)} \parallel^2}{\sum_t \parallel m_t \parallel^2}, \tag{S6}$$

where $\varepsilon_t^{(k)} \equiv m_t - \sum_i^k \beta_i\gamma_{it}$ is the error associated with the specification using k principal components. In the LC model $k = 1$.

The value of k_t in Eq (S5) is adjusted in a second stage to fit the reported values of the observed life expectancy at birth from the observed period [7]. To forecast the k_t values into the future, the LC model uses a random walk with drift model, as follows:

$$k_t = k_{t-1} + c + e_t\sigma, \tag{S7}$$

where c is a drift term that represents the linear trend component in the change of k_t, and $e_t\sigma$ represents random fluctuations in this linear change [6]. The drift term is estimated using the following expression:

$$\hat{c} = \frac{k_{u_T} - k_{u_0}}{u_T - u_0}, \tag{S8}$$

where u_0, u_1, ..., u_T, represent times with gaps, and the error term in Eq (S7) is estimated as follows:

$$\hat{\sigma}^2 \approx \frac{\sum_{t=1}^{T}[k_{u_t} - k_{u_{t-1}} - \hat{c}(u_t - u_{t-1})]^2}{u_T - u_0 - \frac{\sum_{t=1}^{T}[u_t - u_{t-1}]^2}{u_T - u_0}}, \tag{S9}$$

Both the underlying variation of $\hat{c}$ and $\hat{\sigma}$ resulted from incomplete information is considered in the projected values of Eq (S7) and the corresponding mortality projections obtained from Eq (S5). To get a deviation from the linear change of k_t, more than two years of data are necessary for the LC model to provide uncertainty forecasts – by getting positive values of $\hat{\sigma}^2$ in Eq (S9). More details on the estimation of the forecasting values and uncertainty can be found in [6]. In our study, we refer to this modified LC approach as Li-Lee-Tuljapurkar model (LLT).

Annual Reduction Rates

In this study, we measure mortality change using the Average Annual Reduction Rate (ARR), which considers the decline of mortality during a period separated by n years (r_t,r_{t+n}):

$$ARR = 1 - (\frac{r_{t+n}}{r_t})^{\frac{1}{n}}, \tag{S10}$$

where r_t and r_{t+n} are mortality rates at time t and $t+n$, respectively, and n is the number of years between t and $t+n$.

References

1. Greville TNE. Short Methods of Constructing Life Tables. Record of the American Institute of Actuaries. 1943;32:29–42.

2. Chiang C-L. An Introduction to Stochastic Processes in Biostatistics. New York: Wiley; 1968.

3. Camarda CG. **MortalitySmooth**: An R Package for Smoothing Poisson Counts with P-Splines. J Stat Softw [Internet]. 2012 [cited 2017 May 20];50(1). Available from: http://www.jstatsoft.org/v50/i01/.

4. Currie ID, Durban M, Eilers PHC. Generalized Linear Array Models with Applications to Multidimensional Smoothing. J R Stat Soc Ser B Stat Methodol. 2006;68(2):259–80.

5. Eilers PHC, Marx BD. Flexible smoothing with B-splines and penalties. Stat Sci. 1996 May;11(2):89–121.

6. Li N, Lee R, Tuljapurkar S. Using the Lee-Carter Method to Forecast Mortality for Populations with Limited Data. Int Stat Rev Rev Int Stat. 2004;72(1):19–36.

7. Lee R, Miller T. Evaluating the performance of the Lee-Carter method for forecasting mortality. Demography. 2001;38(4):537–549.
