# Supplementary material for: Age distribution, trends, and forecasts of under-5 mortality in 31 sub-Saharan African countries: A modeling study
Source: PLoS Med. 2019 Mar 12;16(3):e1002757. doi: 10.1371/journal.pmed.1002757 (PMC6413894; doi:10.1371/journal.pmed.1002757)
Supplement: S1 Table — A brief note with formula to estimate VE is in S1 Text. Highlighted years correspond to the DHS type and year available for this analysis. For Liberia, the latest survey year was not used for analysis but to construct retrospective years for analysis. ARR, annual rate of reduction; DHS, Demographic and Health Survey; IMR, infant mortality rate; NMR, neonatal mortality rate; U5MR, under-5 mortality rate; UN IGME, United Nations Inter-agency Group for Child Mortality Estimation; VE, variance explained. (PDF) [file pmed.1002757.s003.pdf]

Country	VE_{unigme}	VE_{DHS}	Year with estimated age patterns of mortality																											
Angola	92	89		1991					1996					2001					2006					2011					2016	
Benin	97	90	1990	1991	1992	1993	1994	1995	1996	1997	1998	1999	2000	2001	2002	2003	2004	2005	2006	2007	2008	2009	2010	2011	2012					
Burkina Faso	94	90	1990	1991	1992	1993	1994	1995	1996	1997	1998	1999	2000	2001	2002	2003	2004	2005	2006	2007	2008	2009	2010							
Burundi	93	91	1990			1993			1996			1999			2002			2005			2008			2011	2012					2017
Cameroon	96	92	1990	1991	1992	1993	1994	1995	1996	1997	1998	1999	2000	2001	2002	2003	2004	2005	2006	2007	2008	2009	2010	2011						
Chad	95	93	1990	1991	1992	1993	1994	1995	1996	1997	1998	1999	2000	2001	2002	2003	2004	2005		2007		2009		2011		2013		2015		
Congo	92	89				1993	1994		1996	1997		1999	2000		2002	2003		2005	2006			2009			2012					
Cote d'Ivoire	96	93	1990		1992	1993	1994		1996		1998	1999	2000		2002		2004		2006		2008		2010		2012					
DR Congo	94	91		1991	1992	1993	1994	1995	1996	1997	1998	1999	2000	2001	2002	2003	2004	2005	2006	2007	2008		2010		2012		2014			
Ethiopia	96	94	1990	1991	1992	1993	1994	1995	1996	1997	1998	1999	2000	2001	2002	2003	2004	2005	2006	2007	2008	2009	2010	2011	2012	2013	2014	2015	2016	
Gabon	95	90			1992			1995			1998		2000	2001		2003			2006			2009			2012					
Gambia	94	87				1993					1998					2003					2008					2013				
Ghana	96	94		1991		1993	1994	1995		1997	1998	1999	2000			2003					2008	2009					2014			
Guinea	93	89	1990	1991	1992	1993	1994	1995	1996	1997	1998	1999	2000	2001	2002	2003	2004	2005	2006	2007	2008	2009	2010	2011	2012					
Kenya	95	92	1990	1991	1992	1993	1994		1996		1998		2000		2002		2004				2008		2010		2012		2014			
Lesotho	97	96						1995		1997		1999		2001	2002	2003		2005		2007	2008		2010							
Liberia	94	89		1991		1993		1995		1997		1999		2001		2003		2005		2007		2009		2011		2013				
Malawi	96	92	1990	1991	1992	1993	1994	1995	1996	1997	1998	1999	2000	2001	2002	2003	2004	2005	2006		2008		2010		2012		2014		2016	
Mali	95	93	1990	1991	1992	1993	1994	1995	1996	1997	1998	1999	2000	2001	2002	2003	2004	2005	2006	2007	2008	2009	2010	2011	2012					
Mozambique	94	91	1990	1991	1992	1993	1994	1995	1996	1997	1998	1999	2000	2001	2002	2003	2004	2005	2006	2007	2008	2009	2010	2011						
Namibia	91	86			1992			1995			1998			2001			2004			2007										
Niger	81	80	1990	1991	1992	1993	1994	1995	1996	1997	1998	1999	2000	2001	2002	2003	2004	2005	2006	2007	2008	2009	2010	2011	2012					
Nigeria	98	94	1990	1991	1992	1993	1994	1995	1996	1997	1998	1999	2000	2001	2002	2003	2004	2005	2006	2007	2008	2009	2010	2011	2012	2013				
Rwanda	93	91					1994	1995	1996	1997	1998	1999	2000	2001	2002	2003	2004	2005	2006	2007	2008	2009	2010	2011	2012	2013	2014			
Senegal	97	94	1990	1991	1992	1993	1994	1995	1996	1997	1998	1999	2000	2001	2002	2003	2004	2005	2006	2007	2008	2009	2010	2011	2012	2013	2014	2015		2017
Sierra Leone	94	93				1993					1998					2003					2008					2013				
Togo	94	93	1990		1992		1994		1996		1998		2000		2002		2004		2006		2008				2012		2014			
Uganda	93	90	1990	1991	1992	1993	1994	1995	1996	1997	1998	1999	2000	2001	2002	2003	2004	2005	2006	2007	2008	2009	2010	2011					2016	
Tanzania	95	92	1990	1991	1992	1993	1994	1995	1996	1997	1998	1999		2001		2003	2004	2005		2007			2010			2013			2016	
Zambia	96	93	1990	1991	1992	1993	1994	1995	1996	1997	1998	1999	2000	2001	2002	2003	2004	2005	2006	2007	2008		2010		2012		2014			
Zimbabwe	96	90	1990	1991			1994	1995		1997		1999	2000			2003		2005	2006		2008	2009		2011	2012					

Standard DHS
Continuous DHS
