# Supplementary figures and images for: Age distribution, trends, and forecasts of under-5 mortality in 31 sub-Saharan African countries: A modeling study
Source: PLoS Med. 2019 Mar 12;16(3):e1002757. doi: 10.1371/journal.pmed.1002757 (PMC6413894; doi:10.1371/journal.pmed.1002757)

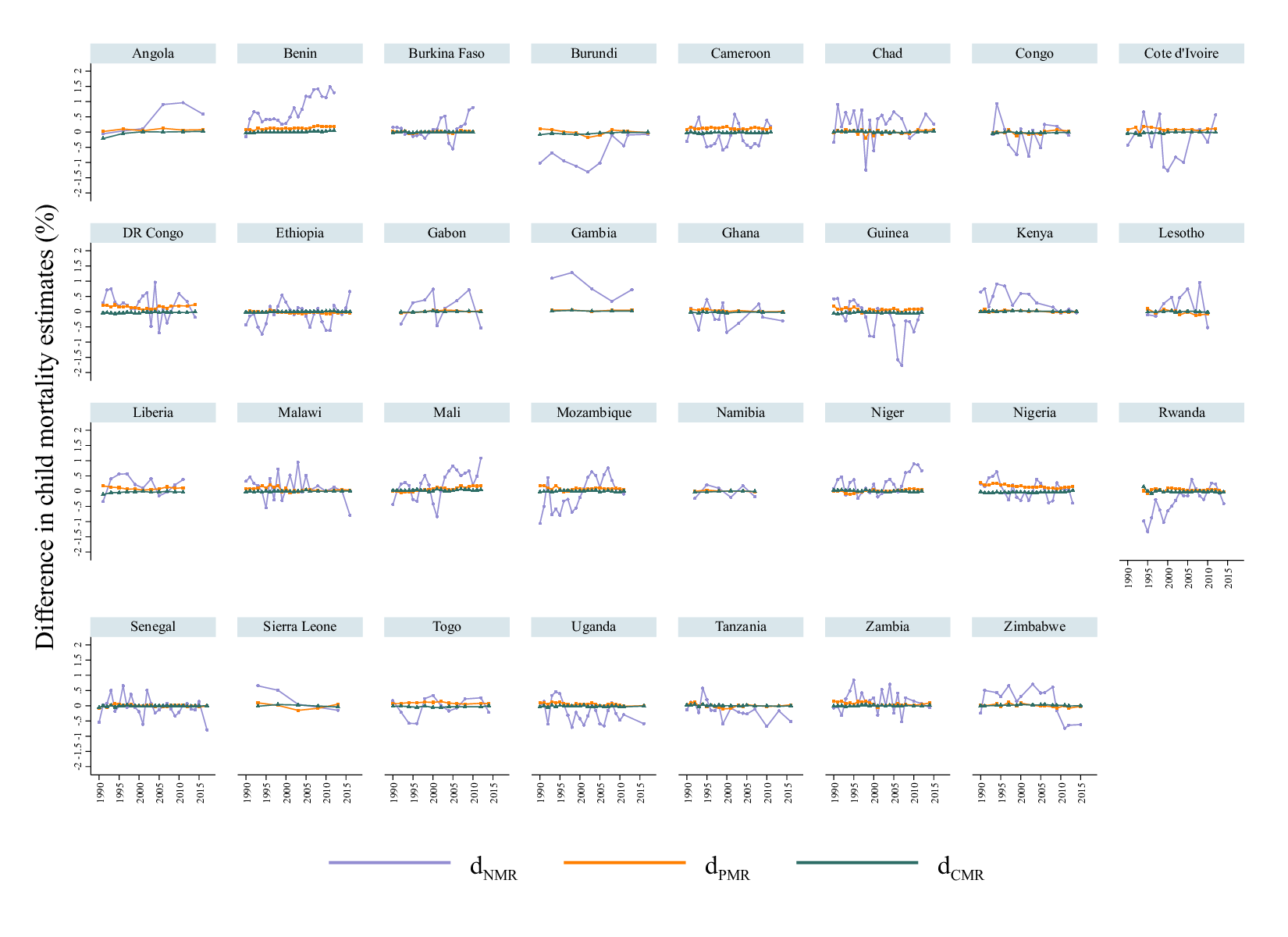

Supplement: S1 Fig — Authors’ estimates using data from the DHS Program. PMR is the probability of dying between 1 month (28 days) and 11 months of age, expressed per 1,000 live births, and the CMR is the probability of dying between 1 and 4 years of age, expressed per 1,000 children age 1 [4]. CMR, child mortality rate; DHS, Demographic and Health Survey; PMR, postneonatal mortality rate; SSA, sub-Saharan Africa; UN IGME, United Nations Inter-agency Group for Child Mortality Estimation. (TIFF) [file pmed.1002757.s004.tiff]

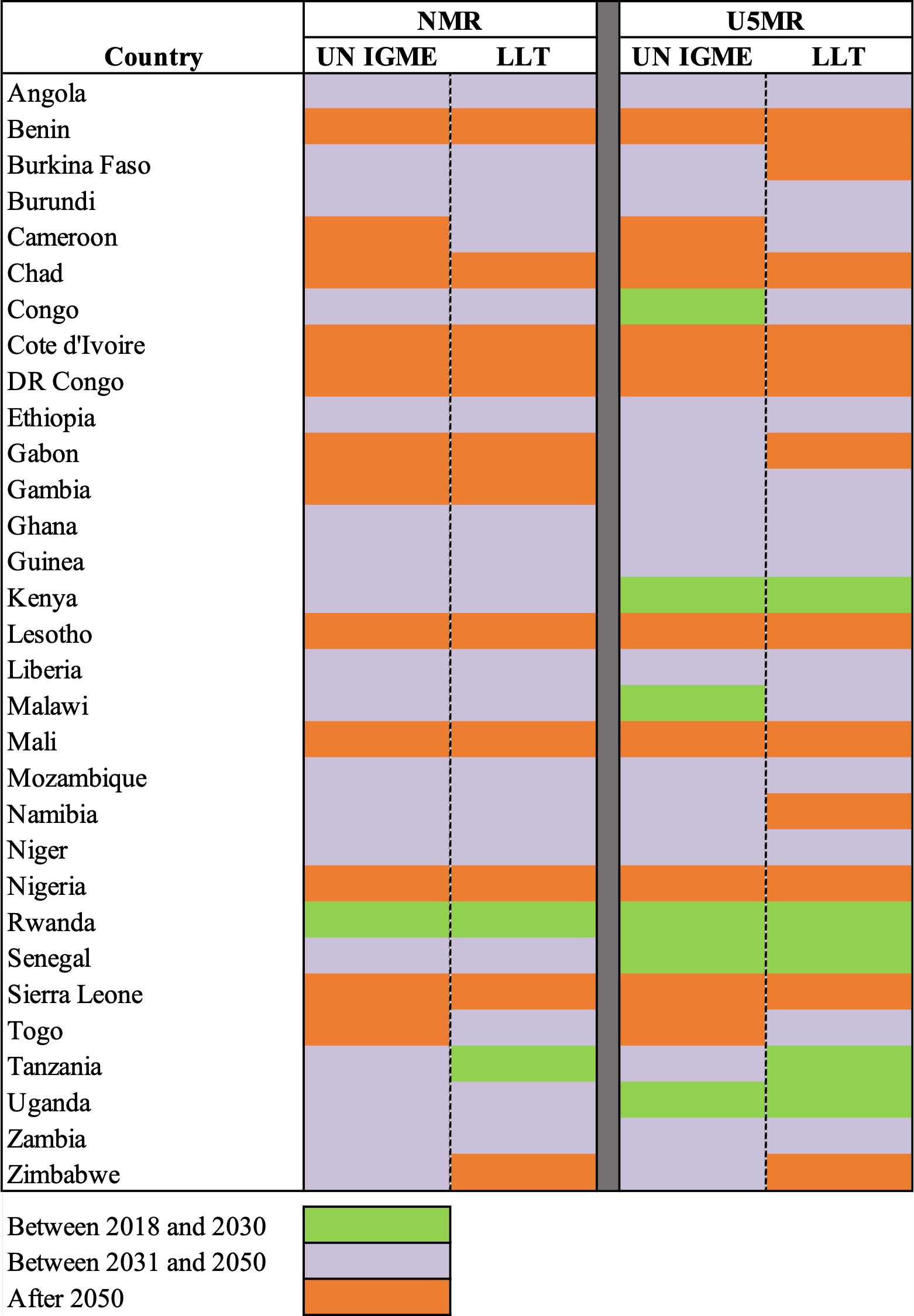

Supplement: S2 Fig — In the LLT, we report wide error bounds for our prediction models for 2030 and 2050. We retrieved UN IGME estimates online from http://data.unicef.org/topic/child-survival/child-survival-sdgs/#. LLT, Li–Lee–Tuljapurkar; NMR, neonatal mortality rate; SDG-3, Sustainability Goal 3; SSA, sub-Saharan Africa; U5MR, under-5 mortality rate; UN IGME, United Nations Inter-agency Group for Child Mortality Estimation. (TIF) [file pmed.1002757.s005.tif]

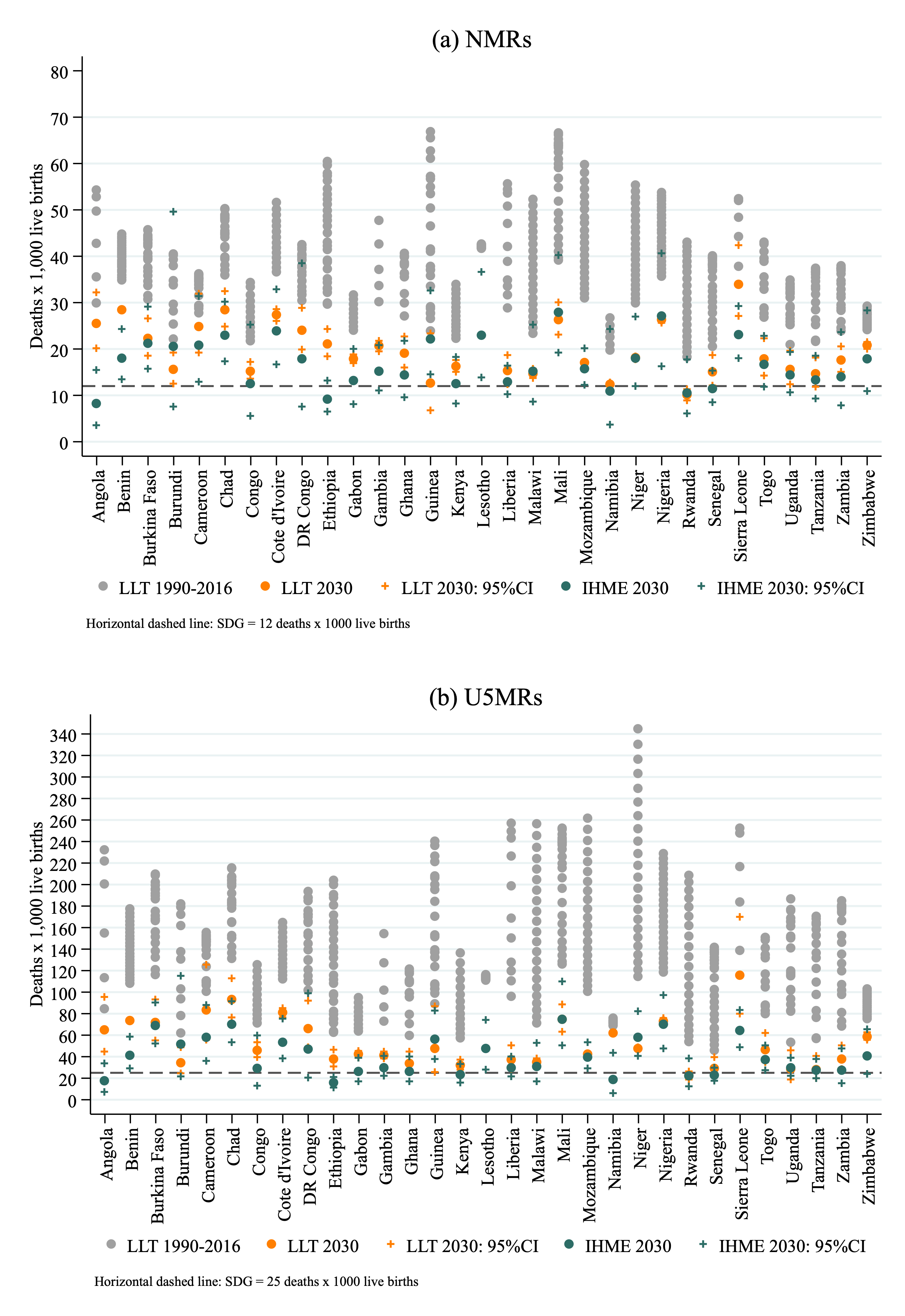

Supplement: S3 Fig — (a) NMRs and (b) U5MRs. In LLT, predictions for Lesotho were precluded by the poor quality of data and great uncertainty in the estimates and uncertainties, and we report unbiased error bounds for our prediction models for 2030. We retrieved IHME estimates online from https://vizhub.healthdata.org/sdg/. IHME, Institute for Health Metrics and Evaluation; LLT, Li–Lee–Tuljapurkar; SDG-3, Sustainability Development Goal 3; SSA, sub-Saharan Africa; U5MR, under-5 mortality rate. (TIF) [file pmed.1002757.s006.tif]
